# Nutrition and Developmental Origins of Kidney Disease

**DOI:** 10.3390/nu15194207

**Published:** 2023-09-29

**Authors:** Long T. Nguyen, Carol A. Pollock, Sonia Saad

**Affiliations:** Renal Research Group, Kolling Institute, St. Leonards, NSW 2065, Australia; carol.pollock@sydney.edu.au (C.A.P.); sonia.saad@sydney.edu.au (S.S.)

**Keywords:** nutrition, development, fetal programming, kidney disease

## Abstract

The developmental programming hypothesis proposes that adverse environmental insults during critical developmental periods increase the risk of diseases later in life. The kidneys are deemed susceptible to such a process, although the exact mechanisms remain elusive. Many factors have been reported to contribute to the developmental origin of chronic kidney diseases (CKD), among which peri-gestational nutrition has a central role, affecting kidney development and metabolism. Physiologically, the link between malnutrition, reduced glomerular numbers, and increased blood pressure is key in the developmental programming of CKD. However, recent studies regarding oxidative stress, mitochondrial dysfunction, epigenetic modifications, and metabolic changes have revealed potential novel pathways for therapeutic intervention. This review will discuss the role of imbalanced nutrition in the development of CKD.

## 1. Introduction

Developmental programming is a mechanism, whereby health complications in adulthood are attributed to physiological stress that occurs during or even before gestation [1,2]. This concept was originally proposed by Barker et al., whose studies showed that infant mortality rates and childhood nutrition in the 1920s correlated significantly with the rate of ischemic heart disease in the 1970s [3,4,5], suggesting that poor nutrition during pregnancy/early life can increase the risk of cardiovascular diseases in the offspring. Since then, extensive evidence supporting the hypothesis has been reported, showing the detrimental effect of different stimuli during gestation, such as stress, smoke exposure, hypertension, and metabolic disorders, on the offspring, which can persist over multiple generations [6,7]. Adverse effects during the perinatal period were also shown to predispose the offspring to a number of metabolic and chronic diseases, such as neurological disorders, fatty liver disease, as well as cardiovascular and chronic kidney diseases (CKDs) [8,9,10,11,12].

CKD is evident in approximately 10% of the global population and characterized by a drop in the estimated glomerular filtration rate (eGFR) below 60 mL/min/1.73 m^2^ and/or presence of protein in the urine for at least 3 months [13]. Hypertension and diabetes mellitus in combination account for over 50% of kidney failure, suggesting the importance of haemodynamic and metabolic regulation in nephropathy. At the cellular/molecular levels, CKD is driven by dysregulated inflammatory and fibrotic pathways, involving complex crosstalk of injured kidney cells with fibroblasts and immune cells via paracrine and autocrine signalling. Initial protective responses to renal injury can result in irreversible progressive kidney damage if they become sustained and, thus, maladaptive. Recurring oxidative stress and inflammation signals induce renal fibroblasts and epithelial cells to differentiate into myofibroblasts, leading to excessive deposition of extracellular matrix (ECM) proteins, such as fibronectin, elastin, and collagens [14]. As such, functional kidney tissue is replaced by fibrous connective tissue with excessive ECM deposition. The presence of interstitial fibrosis is a histological hallmark of CKD, which closely correlates with functional renal decline [15]. To date, it is still unclear why certain patients are more susceptible or, conversely, resistant to the development of CKD. It is hypothesised that such differences are, at least in part, attributed to factors occurring in utero development.

In humans, nephrogenesis starts from the thỉrd week of embryonic development and is generally completed by week 36 of gestation [16]. Therefore, pre-term birth (<gestational week 37) is associated with a risk of reduced kidney size and nephron number in infants and young children (5–7 years old). Additionally, a low nephron number is a known risk factor for high blood pressure in adults [17]. It was hypothesized by Brenner et al. in the late 19th century that low birth weight contributes to hypertension and susceptibility to CKD in adults [18]. While this concept remains central to the developmental origin of CKD, no clinical studies have unequivocally shown that nephron endowment mediates CKD and hypertension. Meanwhile, other processes, such as metabolic dysregulation, oxidative stress, mitochondrial dysfunction, and, especially, epigenetic modification, have emerged as important pathways in CKD development that can be therapeutically targeted. Herein, we summarise the mainstream concept but will be focusing on these alternative pathways.

## 2. General Models in Developmental Programming

The cumulative stress model is a central concept in the developmental origin of health and disease (DoHaD). It assumes that the adverse intrauterine environment has a cumulative effect on foetal growth and, concomitantly, the risk of chronic diseases in adulthood. This theory emphasizes the importance of cumulative insults, whereby the higher intensity or frequency of the intrauterine stress exposure, the higher risk of developmental and functional incompetency [19]. This is because impairment at critical windows of foetal development can render irreversible structural and functional defects [20] that may be magnified by further insults across the lifespan. For instance, rat offspring exposed to malnutrition, both prenatally and during lactation, had lower body weight and reduced energy intake but higher blood glucose levels than those exposed to malnutrition prenatally only [21]. On the other hand, a postnatal high-fat diet (HFD) exacerbated kidney inflammation and fibrosis in offspring mice born to obese dams [22]. HFD consumption in both F0 and F1 generations cumulatively induces microalbuminuria and kidney fibrosis in female F2 offspring, an effect that was partially attributed to DNA methylation modification [23].

In contrast to the cumulative stress model, the predictive adaptive responses and human evolution models (match–mismatch model) explain how foetuses are programmed according to the prenatal environment in expectation that the postnatal environment will remain unchanged [24]. When an intrauterine nutrition imbalance occurs, developmental plasticity allows foetal metabolism and tissue development to be altered in a way that maximises postnatal survival in the same nutrition conditions as in utero. If the eventual mature environment, whether subject to overnutrition or undernutrition, matches the anticipation, then the risk of chronic diseases later in life is reduced. Conversely, if a mismatch occurs, particularly when the postnatal environment has improved, then all the preconditioned changes to growth and metabolism will backfire, leading to suboptimal responses to the environment (e.g., diet or lifestyle) and, hence, structural/functional disorders. Sheep offspring born to nutrient-restricted dams and then weaned on an obesogenic diet demonstrated a significantly higher level of renal lipid accumulation at 1 year of age [25].

Developmental programming is a highly complex process, and whether it occurs primarily through cumulative stress or predictive adaptive responses depends on various factors, including species (e.g., human vs. rodents), genetic (i.e., different mouse strains), the nature and intensity of insults (i.e., dietary composition and length), tissue type (e.g., liver vs. kidney), and also sex (males vs. females). Rats tend to be more susceptible to obesity than mice [26], and according to our experience, a mixed high-fat, high-fructose diet has a stronger effect on weight gain and diabetes in mice than a pure high-fat diet. Regarding tissue specificity, maternal HFD induced a cumulative effect on offspring kidney injury [22,27] but not fatty liver disease or hypertension [22,27,28].

Sex-dependent differences in developmental programming are a well-recognised phenomenon [29], predominantly as a result of intrauterine undernutrition [21,30,31] but also, to a lesser extent, overnutrition [32]. Regarding the kidneys, females tend to have ~12–17% fewer nephrons than males, likely attributed to the lower birth weight [33]. While foetal growth retardation (FGR) can equally affect both male and female offspring, leading to nephron deficits [34], renal dysfunction was only found in males but not females in adulthood. Furthermore, Ryan et al. demonstrated that, in general, female infants, but not males, exhibited glomerular hypertrophy from mid-gestation to term [16], which can compensate for the lower nephron endowment in females and potentially protect females from future kidney disease. On the other hand, it has been argued that such intrarenal compensation to maintain the normal glomerular filtration rate can lead to systemic and glomerular hypertension and, hence, a higher risk of progressive CKD [35]. Regarding intrauterine overnutrition, a maternal HFD has been shown to induce insulin resistance and pancreatic β-cell dysfunction in male offspring only. Similarly, we also showed that, maternal HFD feeding in mice induced changes in renal lipid metabolism and stress responses that were specific to male offspring [36]. In the same study, male offspring kidneys also showed reduced expression of Sirtuin (SIRT)1, of which the function in DoHaD has been reviewed previously [2]. In rats, a maternal high-fat diet led to glomerulosclerosis and impaired renal function, though only in male offspring [37]. Similarly, renal alterations in female offspring exposed to maternal high-fat diet are less severe compared to males [38]. Overall, male offspring appear to be more susceptible to CKD than female offspring when it comes to the developmental programming effects of maternal obesity [39].

## 3. Foetal Growth and Kidney Development

There are approximately 1 million nephrons in normal human kidneys [40]. This number can vary, but a low nephron number is associated with glomerular hypertrophy in certain ethnic groups, such as Australian Aborigines and White Americans [41]. As the nephron number is determined before birth, kidney underdevelopment can have a significant impact on kidney pathophysiology. This topic has been reviewed extensively in the past [33,42,43], and, overall, the evidence demonstrates that developmental programming of CKD and hypertension in preterm and low-birth-weight (LBW) infants is mediated, at least in part, by reduced nephron endowment [33,42,43]. LBW, small for gestational age (SGA), and preterm birth have been associated with an odds ratio for CKD of 1.72, 1.79, and 1.48, respectively, according to a recent meta-analysis involving 2,663,010 Norwegians after a mean follow-up of 26 years [12]. Meanwhile, uteroplacental insufficiency or foetal growth retardation (FGR) has been shown to increase the risk of microalbuminuria, eGFR decline, and kidney failure by 81%, 79%, and 58%, respectively [44]. eGFR but not albuminuria in childhood significantly correlated with maternal protein intake, particularly from vegetables, during the first trimester [45]. Increased incidence of focal segmental glomerulosclerosis (FSGS) was also reported in association with LBW and premature birth [46].

In animals, offspring born to dams fed a protein restriction diet showed a significant reduction in the number of nephrons, coupled with hypertension and glomerular hypertrophy in adulthood [47,48]. In addition, renal apoptosis was found to be upregulated in newborn mice due to maternal food restriction during gestation [49], potentially contributing to the reduced nephron number. In association with the upregulation of proapoptotic genes, maternal protein deficiency also resulted in the downregulation of ciliogenesis factors and cilial elongation in renal tubular epithelial cells in foetal rats [50], which is considered a marker of tubular injury. Although nutrient deprivation is generally considered to be the cause of FGR, maternal obesity has also been associated with higher levels of stillbirth and severe FGR [51,52]. A study in Indigenous Australians, who are twice as likely to develop CKD compared to non-indigenous Australians, showed that babies of obese mothers tend to have reduced kidney size relative to their body weight [53].

## 4. Metabolic Programming

Offspring metabolism is greatly impacted by maternal nutritional status [54,55]. In particular, maternal obesity is one of the strongest risk factors for childhood obesity and comorbidities in the offspring, leading to a vicious cycle that is worsening the global obesity pandemic [56]. Increased levels of glucose and lipids in the maternal bloodstream have been shown to induce metabolic pressure, lipotoxicity, oxidative stress, inflammation, and vasculopathy in the foetal placental unit [57,58,59,60]. The selectivity of the blood–placental barrier was impaired due to maternal obesity together with the upregulation of glucose and fatty acid transporters, leading to an excess of nutrients, metabolic hormones, and proinflammatory cytokines that predispose offspring to metabolic disorders [59,61]. Hyperinsulinaemia and insulin resistance were also induced in the offspring, hence an increased risk for type 2 diabetes and diabetic kidney disease [28,62]. Additionally, higher pre-pregnancy body mass index has been associated with an increased risk for hypertension in the offspring, which can contribute to CKD, although the effect appears to be primarily driven by the offspring nutritional status [63,64].

Maternal obesity is also associated with increased leptin concentration and leptin resistance in the offspring, coupled with dysregulation in appetite control, food preference, and overfeeding. We have also observed that mouse offspring born to HFD-fed dams showed increased milk intake, glucose intolerance, and hyperlipidaemia [28,62]. The hypothalamus, the brain region controlling appetite, showed altered levels of appetite-regulating hormones and neuropeptide receptors [65]. Increased levels of the Melanocortin-4 receptor (Mc4r) in the paraventricular nucleus of the hypothalamus have been shown to mediate hypertension in the offspring born to obese mothers [66]. In the same study, pharmacological inhibition or genetic deletion of Mc4r in the PVH led to the suppression of renin and angiotensin in the renal cortex and reduced blood pressure in the offspring, while restoration of this gene reversed the effect, suggesting interplay between central and renal sympathetic nerve activity and a key role of the brain–kidney axis in the maternal obesity-induced programming of hypertension and CKD. In another study, maternal HFD was associated with baroreflex dysfunction and activation of angiotensin II, leading to hypertension in adult offspring [67]. These programmed changes may serve as the origins of CKD. Indeed, mouse offspring born to HFD-fed dams showed an upregulation in the expression of oxidative stress and inflammatory and fibrotic markers in the kidney [22].

Similar to maternal overnutrition, maternal undernutrition during gestation and lactation has been shown to alter central leptin signalling and the response of Proopiomelanocortin neurons to changes in the energy status. It also increases food intake in adult rats, hence increasing the risk for obesity and related disorders [68,69]. Importantly, caloric restriction during different periods of gestation can lead to different metabolic outcomes in the offspring. While maternal exposure to food restriction during the first half of pregnancy predisposed offspring to a higher risk of obesity, exposure from the third trimester to birth reduced the risk for obesity but increased the incidence of type 2 diabetes mellitus [70]. Such an example demonstrates the complexity in metabolic programming, which contributes to the varied susceptibility to CKD development later in life. As maternal smoking is known to induce FGR, its combination with maternal obesity was shown to exacerbate adiposity and metabolic disorders in male offspring [71], which further predispose them to CKD.

## 5. Oxidative Stress and Mitochondrial Dysfunction

Oxidative stress is a central pathological pathway of many chronic diseases, which is characterised by an overproduction of reactive oxygen species (ROS) and/or a reduction in antioxidant capacity. Such an imbalance increases the level of oxidative damage to cellular components, such as DNA, proteins and lipids, cell cycle arrest, or cell dysfunction [72]. As ROS are by-products of the oxidative phosphorylation process during ATP synthesis by mitochondria, mitochondria are typically vulnerable to oxidative insults. As foetal mitochondria together with mitochondrial DNA are maternally inherited, oxidative damage induced to oocyte mitochondrial DNA can be transmitted to the foetus, initiating disease programming at the very first stage of foetal life. Oxidative stress and/or mitochondrial dysfunction have been implicated in the developmental programming of kidneys in offspring born of dams exposed to diverse conditions inducing nutrition imbalances, such as caloric restriction [73,74], low-protein diet [75], maternal diabetes [76,77], and HFD [22,27,28]. Particularly, oxidative stress and inflammation can occur in reproductive organs due to maternal obesity and hyperglycaemia [78], disturbing oogenesis and leading to poor-quality oocytes [79,80,81,82], which, if fertilised, will likely affect foetal development [83]. Similarly, maternal undernutrition also increased ovarian oxidative stress and reduced ovarian follicle number in adult rat offspring [84], suggesting that the programming effects on reproduction can be transgenerational. In regards to kidney programming, we have demonstrated that mouse and rat offspring exposed to maternal obesity showed higher levels of oxidative stress markers in the kidney at weaning and early adulthood [22]. Similar findings have been reported by other researchers [85,86,87,88,89]. In addition, oxidative disorders were also evident in offspring tissues, including the liver [90,91] and heart [92].

Dietary supplementation of antioxidants has shown protective effects against oxidative stress in offspring due to developmental programming. The supplementation of resveratrol, a naturally occurring antioxidant, in pregnancy partially prevented maternal protein-restriction-induced oxidative stress and metabolic dysfunction in their offspring’s liver in a sex-specific manner [93]. The antioxidant potential of resveratrol is largely mediated by SIRT1, a master regulator of stress responses and senescence [94,95]. The overexpression or pharmacological activation of SIRT1 in the offspring partially reversed metabolic programming from maternal obesity, reflected by lower body weight and fat mass, improved glucose tolerance, and liver damage in the offspring [28,62], which were associated with a reduction in renal oxidative stress and inflammation markers in the offspring [22]. Conversely, early postnatal overfeeding by reducing litter size in mice was found to decrease the expression of SIRT1 and increase cellular senescence in the kidney at weaning [96]. SIRT1 and SIRT3 are also important regulators of mitochondrial function, as they deacetylate and activate peroxisome proliferator-activated receptor-gamma coactivator PGC-1a, a marker of mitochondrial biogenesis, as well as modulating the transcription of other metabolic markers. As SIRTs are therapeutically targetable, this leads to an intervention for renal oxidative stress and mitochondrial dysfunction due to developmental programming.

## 6. Epigenetic Modification

Epigenetic modification plays a central role in developmental programming. DNA methylation, histone acetylation, and micro RNAs can modify chromatin structure and accessibility to transcription factor binding sites, thereby modulating gene expression without changing the DNA sequence per se. As epigenetic factors are influenced by the environment, this allows adverse changes in the utero environment to program gene expression in the foetus. Epigenetic modification is a dynamic, reversible process. Epigenetic changes during a critical window of foetal development may initiate a vicious cycle of dysregulation in the cellular structure and function, increasing the likelihood of disorders later in life. It can also induce “permanent” changes that persist into adulthood and pass down to future generations [97]. In animals, intrauterine overnutrition can result in metabolic dysregulation, tissue inflammation, and mitochondrial impairment in the third generation, even though the second generation was not fed an HFD [55,98,99], suggesting the likelihood of germ cell programming.

### 6.1. DNA Methylation

Among the three main types of epigenetic modifications, DNA methylation is the most stable and, hence, the most studied in disease programming. DNA methylation can either occur passively or actively via the action of DNA methyl transferases Dnmt1, Dnmt3a, and Dnmt3b [100]. While Dnmt1 is responsible for maintaining the original epigenome during cell division, Dnmt3a and 3b induce de novo methylation to DNA. Premature and extreme-birth-weight human newborns have been reported to have an increased level of global DNA methylation that is associated with increased adiposity later in life [101]. This was consistent with our study, which showed increased expression of DNA methyl transferase Dnmt1 and Dnmt3b in adult offspring born to HFD-fed dams [102]. Dnmt1 and the histone methylation marker (H3K27me3) have been implicated in the self-renewal and differentiation of nephron progenitor cells (NPCs) [103]. Dnmt1 has also been suggested to be the key regulator of prenatal renal programming in offspring exposed to maternal protein deficiency [104]. This represents a fundamental link between nephron number, the renin–angiotensin system (RAS), and the intrauterine environment.

In utero exposure to an HFD was associated with hepatic hypermethylation and gene dysregulation with relevance to the development of metabolic syndrome in male mice [105]. Differences in DNA methylation of 3360 loci were identified in this study, among which a great number were associated with transcriptional changes that were maintained into adulthood. In humans, genome-wide correlation of cord blood samples from mother–child pairs led to the identification of specific methylation sites in the offspring of obese mothers that persisted until adulthood [106]. On the other hand, specific changes in DNA methylation profiles from blood and kidney tissue in patients with CKD have been identified in recent studies [107,108,109]. Regarding the developmental programming of CKD, altered methylation of angiotensin receptor 1 (AGTR1) has been shown to mediate the effect of prenatal famine exposure on eGFR decline across consecutive generations in humans [110]. In hypertensive rats, it was demonstrated that maternal protein deficiency in addition to a high-salt diet in the offspring resulted in hypertension that was associated with hypermethylation of prostaglandin E receptor 1 (PTGER1) and hypomethylation of angiotensin II receptor type 2 (AGTR2), respectively, in the kidney [111,112]. Further, AGTR1 hypomethylation in the blood was shown to predict CKD progression in HFD-fed mice in adulthood [113], supporting an important role for the DNA methylation of angiotensin receptors and the developmental origin of CKD. In a different study, FGR was found to downregulate the expression of 11β-Hydroxysteroid dehydrogenase type 2 (11beta-HSD2) in the kidney by altering the binding of transcriptional factor to promoter and DNA/histone methylation [114]. As 11beta-HSD2 regulates renal steroid sensitivity, its suppression has been shown to induce hypertension in both animals and humans [115].

As offspring DNA methylation can be modified, this opens opportunities for targeted therapies to reverse these epigenetic abnormalities. Indeed, it has been shown in stroke-prone spontaneously hypertensive rats that a postnatal high-protein diet can reverse early changes in DNA methylation in offspring due to maternal protein restriction [116]. Collectively, this stresses the important role of DNA methylation in developmental programming due to adverse maternal nutrition.

### 6.2. Histone Acetylation

**Figure 1:**
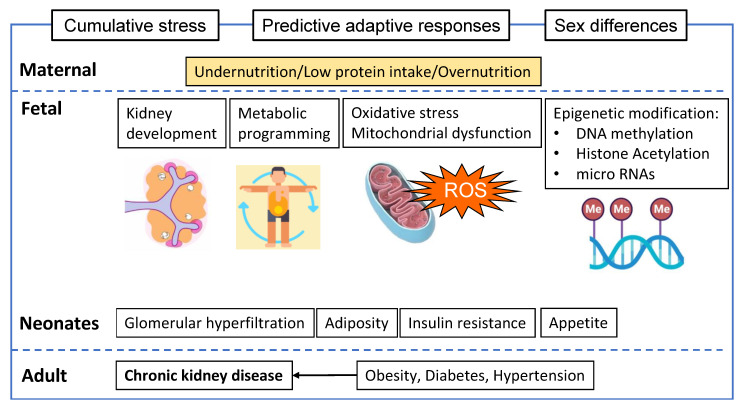
Sub-optimal nutritional conditions in utero induce physiological changes and increase the risk of chronic kidney disease (CKD) later in life. The mechanisms whereby developmental programming affects the kidney are unclear. Apart from the general concepts of cumulative insults and predictive adaptive responses, metabolic programming, oxidative stress, mitochondrial dysfunction, and epigenetic modifications are emerging pathways that can be used for the development of therapeutic interventions.

Histone acetylation is another epigenetic mechanism that is likely involved in developmental programming because it alters DNA coiling and, hence, gene transcription. The foetuses of HFD-fed dams demonstrated histone modifications in the liver that were persistent at postnatal week 5, independent of postnatal feeding [117]. Maternal HFD has been shown to increase foetal hepatic H3K14 acetylation in association with a downregulation of SIRT1 and reduced in vitro deacetylase activity in non-human primates [118]. The effect of maternal undernutrition on histone acetylation and SIRTs in the offspring kidney has not been studied, although, in the liver, uteroplacental insufficiency was shown to alter DNA methylation and histone acetylation in FGR rats [119]. In low-birthweight rat offspring, the postnatal expression of SIRT1 was reduced in association with increased lipogenesis and decreased lipolysis, despite upregulation during the prenatal period [120]. As such, SIRTs are deemed to play a central role in the integrative regulation of metabolic dysregulation, mitochondrial dysfunction, and epigenetic programming [2]. Collectively, the above studies demonstrate a role for in utero metabolic programming. The proposed mechanisms are described in Figure 1.

### 6.3. MicroRNAs

MicroRNAs (miRNAs) are small noncoding RNAs that regulate gene expression through translational repression and mRNA destabilization. Changes in the composition of the miRNA profile are associated with the modulation of diverse biological processes. Particularly, dysregulation of miRNA expression has been implicated in the pathogenesis of developmental kidney diseases [121]. In human, changes in miRNAs involved in oxidative stress, inflammation, and metabolic metabolism were found in the blood of mothers with obesity and gestational diabetes compared to those with normal weight [122]. Similarly, the levels of miRNAs in the placenta and amnion of obese mothers have been shown to be predictors of lower birth weight and increased postnatal weight gain [123,124]. Pathway analysis indicated that these miRNAs were related to insulin signalling and cell proliferation. Consistently, in animals, changes in hepatic and circulating miRNA expression in association with lipid metabolism, insulin signalling, and inflammation due to maternal HFD consumption have been reported [125,126,127]. Rat offspring of HFD-fed dams developed cardiac hypertrophy and increased extracellular matrix deposition in the heart compared to those exposed to a chow diet [128]. These changes were associated with a downregulation of a subset of miRNAs involved in TGFβ.

Regarding the developmental origin of CKD, miRNAs such as miRNA-128 have been shown to induce apoptosis in human embryonic kidney cells [124]. On the other hand, maternal protein restriction altered miRNA expression in foetal and offspring kidneys in rats in association with reduced nephron numbers [129]. Particularly, miR-192 and miR-200 family expression was upregulated in the kidney of rat adult male offspring in association with increased expression of markers of epithelial-to-mesenchymal transition and kidney fibrosis including TGF-β [130,131]. The role of miRNA in maternal obesity-induced kidney programming has not been studied. This clinical and preclinical evidence supports the role of miRNAs in metabolic programming and kidney development, which are important factors for future kidney diseases.

## 7. Conclusions

This review summarized evidence and concepts surrounding the DoHaD theory, which highlights how adverse environmental factors during gestation and lactation can predispose offspring to chronic diseases, particularly CKD later in life. We found that, at the population level, broad mechanisms, such as cumulative stress, predictive adaptive responses, and sex differences, likely formulate the overall developmental programming response of the impacted cohort. At individual and physiological levels, the link between birth weight, glomerular endowment, and hypertension remains the most recognised pathway for the future development of CKD. At the cellular and molecular levels, mechanisms, such as metabolic programming, oxidative stress, mitochondrial dysfunction, and epigenetic modification, are of increasing interest due to their therapeutic targetability. Although a number of studies in animals demonstrate that intervention during gestation, for example, maternal supplementation of antioxidants, can reduce the adverse effects on kidneys and prevent the future development of CKD in the offspring, it remains unclear whether these approaches are beneficial in humans. Due to the chronic nature of CKD, validating these effects in humans requires longitudinal, multi-institutional, and multigenerational studies, which are highly resource-demanding, yet the only option to confirm and intervene in the vicious cycle of metabolic and kidney diseases due to nutrition and developmental programming.

## Data Availability

Not applicable.

## References

[1] Gillman M.W. (2005). Developmental origins of health and disease. N. Engl. J. Med..

[2] Nguyen L.T., Chen H., Pollock C.A., Saad S. (2016). Sirtuins-mediators of maternal obesity-induced complications in offspring?. FASEB J. Off. Publ. Fed. Am. Soc. Exp. Biol..

[3] Barker D.J., Osmond C. (1986). Infant mortality, childhood nutrition, and ischaemic heart disease in England and Wales. Lancet.

[4] Barker D.J., Winter P.D., Osmond C., Margetts B., Simmonds S.J. (1989). Weight in infancy and death from ischaemic heart disease. Lancet.

[5] Brenner B.M., Garcia D.L., Anderson S. (1988). Glomeruli and blood pressure. Less of one, more the other?. Am. J. Hypertens..

[6] Drake A.J., Walker B.R. (2004). The intergenerational effects of fetal programming: Non-genomic mechanisms for the inheritance of low birth weight and cardiovascular risk. J. Endocrinol..

[7] Lahti-Pulkkinen M., Bhattacharya S., Räikkönen K., Osmond C., Norman J.E., Reynolds R.M. (2018). Intergenerational Transmission of Birth Weight Across 3 Generations. Am. J. Epidemiol..

[8] Hellemans K.G., Sliwowska J.H., Verma P., Weinberg J. (2010). Prenatal alcohol exposure: Fetal programming and later life vulnerability to stress, depression and anxiety disorders. Neurosci. Biobehav. Rev..

[9] Borengasser S.J., Kang P., Faske J., Gomez-Acevedo H., Blackburn M.L., Badger T.M., Shankar K. (2014). High fat diet and in utero exposure to maternal obesity disrupts circadian rhythm and leads to metabolic programming of liver in rat offspring. PLoS ONE.

[10] Cleal J.K., Poore K.R., Boullin J.P., Khan O., Chau R., Hambidge O., Torrens C., Newman J.P., Poston L., Noakes D.E. (2007). Mismatched pre- and postnatal nutrition leads to cardiovascular dysfunction and altered renal function in adulthood. Proc. Natl. Acad. Sci. USA.

[11] Catalano P. (2015). Maternal obesity and metabolic risk to the offspring: Why lifestyle interventions may have not achieved the desired outcomes. Int. J. Obes..

[12] Gjerde A., Reisæter A.V., Skrunes R., Marti H.P., Vikse B.E. (2020). Intrauterine Growth Restriction and Risk of Diverse Forms of Kidney Disease during the First 50 Years of Life. CJASN.

[13] Sundström J., Bodegard J., Bollmann A., Vervloet M.G., Mark P.B., Karasik A., Taveira-Gomes T., Botana M., Birkeland K.I., Thuresson M. (2022). Prevalence, outcomes, and cost of chronic kidney disease in a contemporary population of 2·4 million patients from 11 countries: The CaReMe CKD study. Lancet Reg. Health–Eur..

[14] Charlton A., Garzarella J., Jandeleit-Dahm K.A.M., Jha J.C. (2021). Oxidative Stress and Inflammation in Renal and Cardiovascular Complications of Diabetes. Biology.

[15] Misra P.S., Szeto S.G., Krizova A., Gilbert R.E., Yuen D.A. (2020). Renal histology in diabetic nephropathy predicts progression to end-stage kidney disease but not the rate of renal function decline. BMC Nephrol..

[16] Ryan D., Sutherland M.R., Flores T.J., Kent A.L., Dahlstrom J.E., Puelles V.G., Bertram J.F., McMahon A.P., Little M.H., Moore L. (2018). Development of the Human Fetal Kidney from Mid to Late Gestation in Male and Female Infants. EBioMedicine.

[17] Keller G., Zimmer G., Mall G., Ritz E., Amann K. (2003). Nephron number in patients with primary hypertension. N. Engl. J. Med..

[18] Brenner B.M., Mackenzie H.S. (1997). Nephron mass as a risk factor for progression of renal disease. Kidney Int. Suppl..

[19] Belsky J., Schlomer G.L., Ellis B.J. (2012). Beyond cumulative risk: Distinguishing harshness and unpredictability as determinants of parenting and early life history strategy. Dev. Psychol..

[20] Watkins A.J., Wilkins A., Cunningham C., Perry V.H., Seet M.J., Osmond C., Eckert J.J., Torrens C., Cagampang F.R.A., Cleal J. (2008). Low protein diet fed exclusively during mouse oocyte maturation leads to behavioural and cardiovascular abnormalities in offspring. J. Physiol..

[21] Howie G.J., Sloboda D.M., Vickers M.H. (2012). Maternal undernutrition during critical windows of development results in differential and sex-specific effects on postnatal adiposity and related metabolic profiles in adult rat offspring. Br. J. Nutr..

[22] Nguyen L.T., Mak C.H., Chen H., Zaky A.A., Wong M.G., Pollock C.A., Saad S. (2019). SIRT1 Attenuates Kidney Disorders in Male Offspring Due to Maternal High-Fat Diet. Nutrients.

[23] Zhang X., Hasan A.A., Wu H., Gaballa M.M.S., Zeng S., Liu L., Xie L., Jung T., Grune T., Krämer B.K. (2022). High-fat, sucrose and salt-rich diet during rat spermatogenesis lead to the development of chronic kidney disease in the female offspring of the F2 generation. FASEB J. Off. Publ. Fed. Am. Soc. Exp. Biol..

[24] Gluckman P.D., Hanson M.A., Spencer H.G. (2005). Predictive adaptive responses and human evolution. Trends Ecol. Evol..

[25] Fainberg H.P., Sharkey D., Sebert S., Wilson V., Pope M., Budge H., Symonds M.E. (2013). Suboptimal maternal nutrition during early fetal kidney development specifically promotes renal lipid accumulation following juvenile obesity in the offspring. Reprod. Fertil. Dev..

[26] Dissard R., Klein J., Caubet C., Breuil B., Siwy J., Hoffman J., Sicard L., Ducassé L., Rascalou S., Payre B. (2013). Long Term Metabolic Syndrome Induced by a High Fat High Fructose Diet Leads to Minimal Renal Injury in C57BL/6 Mice. PLoS ONE.

[27] Aliou Y., Liao M.-C., Zhao X.-P., Chang S.-Y., Chenier I., Ingelfinger J.R., Zhang S.-L. (2016). Post-weaning high-fat diet accelerates kidney injury, but not hypertension programmed by maternal diabetes. Pediatr. Res..

[28] Nguyen L.T., Chen H., Zaky A., Pollock C., Saad S. (2019). SIRT1 overexpression attenuates offspring metabolic and liver disorders as a result of maternal high-fat feeding. J. Physiol..

[29] Aiken C.E., Ozanne S.E. (2013). Sex differences in developmental programming models. Reproduction.

[30] McMullen S., Langley-Evans S.C. (2005). Sex-specific effects of prenatal low-protein and carbenoxolone exposure on renal angiotensin receptor expression in rats. Hypertension.

[31] Gallo L.A., Tran M., Cullen-McEwen L.A., Denton K.M., Jefferies A.J., Moritz K.M., Wlodek M.E. (2014). Transgenerational programming of fetal nephron deficits and sex-specific adult hypertension in rats. Reprod. Fertil. Dev..

[32] Thone-Reineke C., Kalk P., Dorn M., Klaus S., Simon K., Pfab T., Godes M., Persson P., Unger T., Hocher B. (2006). High-protein nutrition during pregnancy and lactation programs blood pressure, food efficiency, and body weight of the offspring in a sex-dependent manner. Am. J. Physiol. Regul. Integr. Comp. Physiol..

[33] Luyckx V.A., Shukha K., Brenner B.M. (2011). Low nephron number and its clinical consequences. Rambam Maimonides Med. J..

[34] Schreuder M.F., Nyengaard J.R., Fodor M., van Wijk J.A., Delemarre-van de Waal H.A. (2005). Glomerular number and function are influenced by spontaneous and induced low birth weight in rats. J. Am. Soc. Nephrol. JASN.

[35] Bagby S.P. (2007). Maternal Nutrition, Low Nephron Number, and Hypertension in Later Life: Pathways of Nutritional Programming. J. Nutr..

[36] Nguyen L.T., Chen H., Pollock C., Saad S. (2017). SIRT1 reduction is associated with sex-specific dysregulation of renal lipid metabolism and stress responses in offspring by maternal high-fat diet. Sci. Rep..

[37] Jackson C.M., Alexander B.T., Roach L., Haggerty D., Marbury D.C., Hutchens Z.M., Flynn E.R., Maric-Bilkan C. (2012). Exposure to maternal overnutrition and a high-fat diet during early postnatal development increases susceptibility to renal and metabolic injury later in life. Am. J. Physiol. Ren. Physiol..

[38] Flynn E.R., Alexander B.T., Lee J., Hutchens Z.M., Maric-Bilkan C. (2013). High-fat/fructose feeding during prenatal and postnatal development in female rats increases susceptibility to renal and metabolic injury later in life. Am. J. Physiol. Regul. Integr. Comp. Physiol..

[39] Cheong J.N., Wlodek M.E., Moritz K.M., Cuffe J.S. (2016). Programming of maternal and offspring disease: Impact of growth restriction, fetal sex and transmission across generations. J. Physiol..

[40] Bertram J.F., Douglas-Denton R.N., Diouf B., Hughson M.D., Hoy W.E. (2011). Human nephron number: Implications for health and disease. Pediatr. Nephrol..

[41] Douglas-Denton R.N., McNamara B.J., Hoy W.E., Hughson M.D., Bertram J.F. (2006). Does nephron number matter in the development of kidney disease?. Ethn. Dis..

[42] Hoy W.E., Hughson M.D., Bertram J.F., Douglas-Denton R., Amann K. (2005). Nephron Number, Hypertension, Renal Disease, and Renal Failure. J. Am. Soc. Nephrol..

[43] Kanzaki G., Tsuboi N., Haruhara K., Koike K., Ogura M., Shimizu A., Yokoo T. (2015). Factors associated with a vicious cycle involving a low nephron number, hypertension and chronic kidney disease. Hypertens. Res..

[44] Keijzer-Veen M.G., Schrevel M., Finken M.J., Dekker F.W., Nauta J., Hille E.T., Frölich M., van der Heijden B.J. (2005). Microalbuminuria and lower glomerular filtration rate at young adult age in subjects born very premature and after intrauterine growth retardation. J. Am. Soc. Nephrol. JASN.

[45] Miliku K., Voortman T., van den Hooven E.H., Hofman A., Franco O.H., Jaddoe V.W. (2015). First-trimester maternal protein intake and childhood kidney outcomes: The Generation R Study. Am. J. Clin. Nutr..

[46] Ikezumi Y., Suzuki T., Karasawa T., Yamada T., Hasegawa H., Nishimura H., Uchiyama M. (2013). Low birthweight and premature birth are risk factors for podocytopenia and focal segmental glomerulosclerosis. Am. J. Nephrol..

[47] Jones S., Bilous R., Flyvbjerg A., Marshall S. (2001). Intra-uterine environment influences glomerular number and the acute renal adaptation to experimental diabetes. Diabetologia.

[48] Woods L.L., Weeks D.A., Rasch R. (2004). Programming of adult blood pressure by maternal protein restriction: Role of nephrogenesis. Kidney Int..

[49] Tafti S.A., Nast C.C., Desai M., Amaya K.E., Ross M.G., Magee T.R. (2011). Maternal undernutrition upregulates apoptosis in offspring nephrogenesis. J. Dev. Orig. Health Dis..

[50] Wang J., Zhou P., Zhu L., Guan H., Gou J., Liu X. (2023). Maternal protein deficiency alters primary cilia length in renal tubular and impairs kidney development in fetal rat. Front. Nutr..

[51] Radulescu L., Munteanu O., Popa F., Cirstoiu M. (2013). The implications and consequences of maternal obesity on fetal intrauterine growth restriction. J. Med. Life.

[52] Tanner L.D., Brock C., Chauhan S.P. (2022). Severity of fetal growth restriction stratified according to maternal obesity. J. Matern.-Fetal Neonatal Med..

[53] Lee Y.Q., Lumbers E.R., Oldmeadow C., Collins C.E., Johnson V., Keogh L., Sutherland K., Gordon A., Smith R., Rae K.M. (2019). The relationship between maternal adiposity during pregnancy and fetal kidney development and kidney function in infants: The Gomeroi gaaynggal study. Physiol. Rep..

[54] Desai M., Jellyman J.K., Han G., Beall M., Lane R.H., Ross M.G. (2014). Maternal obesity and high-fat diet program offspring metabolic syndrome. Am. J. Obstet. Gynecol..

[55] Saben J.L., Boudoures A.L., Asghar Z., Thompson A., Drury A., Zhang W., Chi M., Cusumano A., Scheaffer S., Moley K.H. (2016). Maternal Metabolic Syndrome Programs Mitochondrial Dysfunction via Germline Changes across Three Generations. Cell Rep..

[56] Gaillard R., Durmuş B., Hofman A., Mackenbach J.P., Steegers E.A., Jaddoe V.W. (2013). Risk factors and outcomes of maternal obesity and excessive weight gain during pregnancy. Obesity.

[57] Liang C., DeCourcy K., Prater M.R. (2010). High–saturated-fat diet induces gestational diabetes and placental vasculopathy in C57BL/6 mice. Metabolism.

[58] Li H.-P., Chen X., Li M.-Q. (2013). Gestational diabetes induces chronic hypoxia stress and excessive inflammatory response in murine placenta. Int. J. Clin. Exp. Pathol..

[59] Zhu M.J., Ma Y., Long N.M., Du M., Ford S.P. (2010). Maternal obesity markedly increases placental fatty acid transporter expression and fetal blood triglycerides at midgestation in the ewe. Am. J. Physiol.-Regul. Integr. Comp. Physiol..

[60] Saben J., Lindsey F., Zhong Y., Thakali K., Badger T.M., Andres A., Gomez-Acevedo H., Shankar K. (2014). Maternal obesity is associated with a lipotoxic placental environment. Placenta.

[61] Jones H.N., Woollett L.A., Barbour N., Prasad P.D., Powell T.L., Jansson T. (2009). High-fat diet before and during pregnancy causes marked up-regulation of placental nutrient transport and fetal overgrowth in C57/BL6 mice. FASEB J..

[62] Nguyen L.T., Chen H., Mak C., Zaky A., Pollock C., Saad S. (2018). SRT1720 attenuates obesity and insulin resistance but not liver damage in the offspring due to maternal and postnatal high-fat diet consumption. Am. J. Physiol.-Endocrinol. Metab..

[63] Brunton N.M., Dufault B., Dart A., Azad M.B., McGavock J.M. (2021). Maternal body mass index, offspring body mass index, and blood pressure at 18 years: A causal mediation analysis. Int. J. Obes..

[64] Eitmann S., Mátrai P., Németh D., Hegyi P., Lukács A., Bérczi B., Czumbel L.M., Kiss I., Gyöngyi Z., Varga G. (2022). Maternal overnutrition elevates offspring’s blood pressure—A systematic review and meta-analysis. Paediatr. Perinat. Epidemiol..

[65] Nguyen L.T., Saad S., Tan Y., Pollock C., Chen H. (2017). Maternal high-fat diet induces metabolic stress response disorders in offspring hypothalamus. J. Mol. Endocrinol..

[66] Samuelsson A.S., Mullier A., Maicas N., Oosterhuis N.R., Eun Bae S., Novoselova T.V., Chan L.F., Pombo J.M., Taylor P.D., Joles J.A. (2016). Central role for melanocortin-4 receptors in offspring hypertension arising from maternal obesity. Proc. Natl. Acad. Sci. USA.

[67] Zhang Y.P., Huo Y.L., Fang Z.Q., Wang X.F., Li J.D., Wang H.P., Peng W., Johnson A.K., Xue B. (2018). Maternal high-fat diet acts on the brain to induce baroreflex dysfunction and sensitization of angiotensin II-induced hypertension in adult offspring. Am. J. Physiol. Heart Circ. Physiol..

[68] Breton C., Lukaszewski M.A., Risold P.Y., Enache M., Guillemot J., Rivière G., Delahaye F., Lesage J., Dutriez-Casteloot I., Laborie C. (2009). Maternal prenatal undernutrition alters the response of POMC neurons to energy status variation in adult male rat offspring. Am. J. Physiol. Endocrinol. Metab..

[69] Qasem R.J., Li J., Tang H.M., Pontiggia L., D’Mello A.P. (2016). Maternal protein restriction during pregnancy and lactation alters central leptin signalling, increases food intake, and decreases bone mass in 1 year old rat offspring. Clin. Exp. Pharmacol. Physiol..

[70] Ravelli G.P., Stein Z.A., Susser M.W. (1976). Obesity in young men after famine exposure in utero and early infancy. N. Engl. J. Med..

[71] Huang T., Yang M., Zeng Y., Huang X., Wang N., Chen Y., Li P., Yuan J., Chen C., Oliver B.G. (2021). Maternal high fat diet consumption exaggerates metabolic disorders in mice with cigarette-smoking induced intrauterine undernutrition. Front. Nutr..

[72] Daenen K., Andries A., Mekahli D., Van Schepdael A., Jouret F., Bammens B. (2019). Oxidative stress in chronic kidney disease. Pediatr. Nephrol..

[73] Tain Y.-L., Hsieh C.-S., Lin I.-C., Chen C.-C., Sheen J.-M., Huang L.-T. (2010). Effects of maternal L-citrulline supplementation on renal function and blood pressure in offspring exposed to maternal caloric restriction: The impact of nitric oxide pathway. Nitric Oxide.

[74] Pereira S.P., Oliveira P.J., Tavares L.C., Moreno A.J., Cox L.A., Nathanielsz P.W., Nijland M.J. (2015). Effects of moderate global maternal nutrient reduction on fetal baboon renal mitochondrial gene expression at 0.9 gestation. Am. J. Physiol. Ren. Physiol..

[75] Engeham S., Mdaki K., Jewell K., Austin R., Lehner A.N., Langley-Evans S.C. (2012). Mitochondrial respiration is decreased in rat kidney following fetal exposure to a maternal low-protein diet. J. Nutr. Metab..

[76] Garcia-Vargas L., Addison S.S., Nistala R., Kurukulasuriya D., Sowers J.R. (2012). Gestational diabetes and the offspring: Implications in the development of the cardiorenal metabolic syndrome in offspring. Cardiorenal Med..

[77] Ornoy A., Reece E.A., Pavlinkova G., Kappen C., Miller R.K. (2015). Effect of maternal diabetes on the embryo, fetus, and children: Congenital anomalies, genetic and epigenetic changes and developmental outcomes. Birth Defects Res. Part C Embryo Today Rev..

[78] Shankar K., Zhong Y., Kang P., Lau F., Blackburn M.L., Chen J.-R., Borengasser S.J., Ronis M.J.J., Badger T.M. (2011). Maternal Obesity Promotes a Proinflammatory Signature in Rat Uterus and Blastocyst. Endocrinology.

[79] Jungheim E.S., Schoeller E.L., Marquard K.L., Louden E.D., Schaffer J.E., Moley K.H. (2010). Diet-induced obesity model: Abnormal oocytes and persistent growth abnormalities in the offspring. Endocrinology.

[80] Shah D.K., Missmer S., Berry K., Racowsky C., Ginsburg E.S. (2010). Oocyte and embryo quality in obese patients undergoing in vitro fertilization (IVF). Fertil. Steril..

[81] Zhang L., Han L., Ma R., Hou X., Yu Y., Sun S., Xu Y., Schedl T., Moley K.H., Wang Q. (2015). Sirt3 prevents maternal obesity-associated oxidative stress and meiotic defects in mouse oocytes. Cell Cycle.

[82] Wang H., Cheng Q., Li X., Hu F., Han L., Zhang H., Li L., Ge J., Ying X., Guo X. (2018). Loss of TIGAR induces oxidative stress and meiotic defects in oocytes from obese mice. Mol. Cell. Proteom..

[83] Han L., Ren C., Li L., Li X., Ge J., Wang H., Miao Y.-L., Guo X., Moley K.H., Shu W. (2018). Embryonic defects induced by maternal obesity in mice derive from Stella insufficiency in oocytes. Nat. Genet..

[84] Bernal A.B., Vickers M.H., Hampton M.B., Poynton R.A., Sloboda D.M. (2010). Maternal undernutrition significantly impacts ovarian follicle number and increases ovarian oxidative stress in adult rat offspring. PLoS ONE.

[85] Glastras S.J., Chen H., McGrath R.T., Zaky A.A., Gill A.J., Pollock C.A., Saad S. (2016). Effect of GLP-1 Receptor Activation on Offspring Kidney Health in a Rat Model of Maternal Obesity. Sci. Rep..

[86] do Nascimento L.C.P., Neto J., de Andrade Braga V., Lagranha C.J., de Brito Alves J.L. (2020). Maternal exposure to high-fat and high-cholesterol diet induces arterial hypertension and oxidative stress along the gut-kidney axis in rat offspring. Life Sci..

[87] Vieira-Filho L.D., Lara L.S., Silva P.A., Luzardo R., Einicker-Lamas M., Cardoso H.D., Paixão A.D., Vieyra A. (2009). Placental oxidative stress in malnourished rats and changes in kidney proximal tubule sodium ATPases in offspring. Clin. Exp. Pharmacol. Physiol..

[88] Pedroza A., Ferreira D.S., Santana D.F., da Silva P.T., de Aguiar Júnior F.C.A., Sellitti D.F., Lagranha C.J. (2019). A maternal low-protein diet and neonatal overnutrition result in similar changes to glomerular morphology and renal cortical oxidative stress measures in male Wistar rats. Appl. Physiol. Nutr. Metab.=Physiol. Appl. Nutr. Et Metab..

[89] Zi Y., Ma C., Li H., Shen S., Liu Y., Li M., Gao F. (2021). Effects of intrauterine growth restriction during late pregnancy on the ovine fetal renal function and antioxidant capacity. Anim. Sci. J.=Nihon Chikusan Gakkaiho.

[90] Xue Y., Guo C., Hu F., Zhu W., Mao S. (2020). Undernutrition-induced lipid metabolism disorder triggers oxidative stress in maternal and fetal livers using a model of pregnant sheep. FASEB J..

[91] Alfaradhi M.Z., Fernandez-Twinn D.S., Martin-Gronert M.S., Musial B., Fowden A., Ozanne S.E. (2014). Oxidative stress and altered lipid homeostasis in the programming of offspring fatty liver by maternal obesity. Am. J. Physiol. Regul. Integr. Comp. Physiol..

[92] Rodríguez-Rodríguez P., López de Pablo A.L., García-Prieto C.F., Somoza B., Quintana-Villamandos B., Gómez de Diego J.J., Gutierrez-Arzapalo P.Y., Ramiro-Cortijo D., González M.C., Arribas S.M. (2017). Long term effects of fetal undernutrition on rat heart. Role of hypertension and oxidative stress. PLoS ONE.

[93] Vega C.C., Reyes-Castro L.A., Rodríguez-González G.L., Bautista C.J., Vázquez-Martínez M., Larrea F., Chamorro-Cevallos G.A., Nathanielsz P.W., Zambrano E. (2016). Resveratrol partially prevents oxidative stress and metabolic dysfunction in pregnant rats fed a low protein diet and their offspring. J. Physiol..

[94] Hubbard B.P., Sinclair D.A. (2014). Small molecule SIRT1 activators for the treatment of aging and age-related diseases. Trends Pharmacol. Sci..

[95] Bitterman K.J., Anderson R.M., Cohen H.Y., Latorre-Esteves M., Sinclair D.A. (2002). Inhibition of silencing and accelerated aging by nicotinamide, a putative negative regulator of yeast sir2 and human SIRT1. J. Biol. Chem..

[96] Juvet C., Siddeek B., Yzydorczyk C., Vergely C., Nardou K., Armengaud J.B., Benahmed M., Simeoni U., Cachat F., Chehade H. (2020). Renal Programming by Transient Postnatal Overfeeding: The Role of Senescence Pathways. Front. Physiol..

[97] Skinner M.K. (2008). What is an epigenetic transgenerational phenotype?: F3 or F2. Reprod. Toxicol..

[98] Tsoulis M.W., Chang P.E., Moore C.J., Chan K.A., Gohir W., Petrik J.J., Vickers M.H., Connor K.L., Sloboda D.M. (2016). Maternal high-fat diet-induced loss of fetal oocytes is associated with compromised follicle growth in adult rat offspring. Biol. Reprod..

[99] Cheong Y., Sadek K.H., Bruce K.D., Macklon N., Cagampang F.R. (2014). Diet-induced maternal obesity alters ovarian morphology and gene expression in the adult mouse offspring. Fertil. Steril..

[100] Li Y., Pollock C.A., Saad S. (2021). Aberrant DNA Methylation Mediates the Transgenerational Risk of Metabolic and Chronic Disease Due to Maternal Obesity and Overnutrition. Genes.

[101] Michels K.B., Harris H.R., Barault L. (2011). Birthweight, maternal weight trajectories and global DNA methylation of LINE-1 repetitive elements. PLoS ONE.

[102] Larkin B.P., Saad S., Glastras S.J., Nguyen L.T., Hou M., Chen H., Wang R., Pollock C.A. (2021). Low-dose hydralazine during gestation reduces renal fibrosis in rodent offspring exposed to maternal high fat diet. PLoS ONE.

[103] Huang B., Liu Z., Vonk A., Zeng Z., Li Z. (2020). Epigenetic regulation of kidney progenitor cells. Stem Cells Transl. Med..

[104] Watanabe I., Jara Z., Volpini R., Franco M.d.C., Jung F., Casarini D. (2018). Up-Regulation of renal renin–Angiotensin system and inflammatory mechanisms in the prenatal programming by low-Protein diet: Beneficial effect of the post-weaning losartan treatment. J. Dev. Orig. Health Dis..

[105] Seki Y., Suzuki M., Guo X., Glenn A.S., Vuguin P.M., Fiallo A., Du Q., Ko Y.-A., Yu Y., Susztak K. (2017). In utero exposure to a high-fat diet programs hepatic hypermethylation and gene dysregulation and development of metabolic syndrome in male mice. Endocrinology.

[106] Sharp G.C., Lawlor D.A., Richmond R.C., Fraser A., Simpkin A., Suderman M., Shihab H.A., Lyttleton O., McArdle W., Ring S.M. (2015). Maternal pre-pregnancy BMI and gestational weight gain, offspring DNA methylation and later offspring adiposity: Findings from the Avon Longitudinal Study of Parents and Children. Int. J. Epidemiol..

[107] Lecamwasam A., Novakovic B., Meyer B., Ekinci E.I., Dwyer K.M., Saffery R. (2020). DNA methylation profiling identifies epigenetic differences between early versus late stages of diabetic chronic kidney disease. Nephrol. Dial. Transplant..

[108] Schlosser P., Tin A., Matias-Garcia P.R., Thio C.H.L., Joehanes R., Liu H., Weihs A., Yu Z., Hoppmann A., Grundner-Culemann F. (2021). Meta-analyses identify DNA methylation associated with kidney function and damage. Nat. Commun..

[109] Dritsoula A., Kislikova M., Oomatia A., Webster A.P., Beck S., Ponticos M., Lindsey B., Norman J., Wheeler D.C., Oates T. (2021). Epigenome-wide methylation profile of chronic kidney disease-derived arterial DNA uncovers novel pathways in disease-associated cardiovascular pathology. Epigenetics.

[110] Jiang W., Han T., Duan W., Dong Q., Hou W., Wu H., Wang Y., Jiang Z., Pei X., Chen Y. (2020). Prenatal famine exposure and estimated glomerular filtration rate across consecutive generations: Association and epigenetic mediation in a population-based cohort study in Suihua China. Aging.

[111] Miyoshi M., Sato M., Saito K., Otani L., Shirahige K., Miura F., Ito T., Jia H., Kato H. (2018). Maternal Protein Restriction Alters the Renal Ptger1 DNA Methylation State in SHRSP Offspring. Nutrients.

[112] Miyoshi M., Imakado Y., Otani L., Kaji M., Aanzai Y., Sugimoto N., Murakami T., Fukuoka M., Hohjoh H., Jia H. (2021). Maternal protein restriction induces renal AT2R promoter hypomethylation in salt-sensitive, hypertensive rats. Food Sci. Nutr..

[113] Nguyen L.T., Larkin B.P., Wang R., Faiz A., Pollock C.A., Saad S. (2022). Blood DNA Methylation Predicts Diabetic Kidney Disease Progression in High Fat Diet-Fed Mice. Nutrients.

[114] Baserga M., Kaur R., Hale M.A., Bares A., Yu X., Callaway C.W., McKnight R.A., Lane R.H. (2010). Fetal growth restriction alters transcription factor binding and epigenetic mechanisms of renal 11beta-hydroxysteroid dehydrogenase type 2 in a sex-specific manner. Am. J. Physiol. Regul. Integr. Comp. Physiol..

[115] Ferrari P., Krozowski Z. (2000). Role of the 11beta-hydroxysteroid dehydrogenase type 2 in blood pressure regulation. Kidney Int..

[116] Ando C., Ma S., Miyoshi M., Furukawa K., Li X., Jia H., Kato H. (2023). Postnatal nutrition environment reprograms renal DNA methylation patterns in offspring of maternal protein-restricted stroke-prone spontaneously hypertensive rats. Front Nutr.

[117] Suter M.A., Ma J., Vuguin P.M., Hartil K., Fiallo A., Harris R.A., Charron M.J., Aagaard K.M. (2014). In utero exposure to a maternal high-fat diet alters the epigenetic histone code in a murine model. Am. J. Obstet. Gynecol..

[118] Suter M.A., Chen A., Burdine M.S., Choudhury M., Harris R.A., Lane R.H., Friedman J.E., Grove K.L., Tackett A.J., Aagaard K.M. (2012). A maternal high-fat diet modulates fetal SIRT1 histone and protein deacetylase activity in nonhuman primates. FASEB J..

[119] MacLennan N.K., James S.J., Melnyk S., Piroozi A., Jernigan S., Hsu J.L., Janke S.M., Pham T.D., Lane R.H. (2004). Uteroplacental insufficiency alters DNA methylation, one-carbon metabolism, and histone acetylation in IUGR rats. Physiol. Genom..

[120] Wolfe D., Gong M., Han G., Magee T.R., Ross M.G., Desai M. (2012). Nutrient sensor–mediated programmed nonalcoholic fatty liver disease in low birthweight offspring. Am. J. Obstet. Gynecol..

[121] Trionfini P., Benigni A., Remuzzi G. (2015). MicroRNAs in kidney physiology and disease. Nat. Rev. Nephrol..

[122] Serati A., Novielli C., Anelli G.M., Mandalari M., Parisi F., Cetin I., Paleari R., Mandò C. (2023). Characterization of Maternal Circulating MicroRNAs in Obese Pregnancies and Gestational Diabetes Mellitus. Antioxidants.

[123] Nardelli C., Iaffaldano L., Ferrigno M., Labruna G., Maruotti G.M., Quaglia F., Capobianco V., Di Noto R., Del Vecchio L., Martinelli P. (2014). Characterization and predicted role of the microRNA expression profile in amnion from obese pregnant women. Int. J. Obes..

[124] Carreras-Badosa G., Bonmatí A., Ortega F.J., Mercader J.M., Guindo-Martínez M., Torrents D., Prats-Puig A., Martinez-Calcerrada J.M., de Zegher F., Ibáñez L. (2017). Dysregulation of Placental miRNA in Maternal Obesity Is Associated With Pre- and Postnatal Growth. J. Clin. Endocrinol. Metab..

[125] Benatti R.O., Melo A.M., Borges F.O., Ignacio-Souza L.M., Simino L.A., Milanski M., Velloso L.A., Torsoni M.A., Torsoni A.S. (2014). Maternal high-fat diet consumption modulates hepatic lipid metabolism and microRNA-122 (miR-122) and microRNA-370 (miR-370) expression in offspring. Br. J. Nutr..

[126] Zheng J., Zhang Q., Mul J.D., Yu M., Xu J., Qi C., Wang T., Xiao X. (2016). Maternal high-calorie diet is associated with altered hepatic microRNA expression and impaired metabolic health in offspring at weaning age. Endocrine.

[127] Gaytán-Pacheco N., Lima-Rogel V., Méndez-Mancilla A., Escalante-Padrón F., Toro-Ortíz J.C., Jiménez-Capdeville M.E., Zaga-Clavellina V., Portales-Pérez D.P., Noyola D.E., Salgado-Bustamante M. (2021). Changes in PPAR-γ Expression Are Associated with microRNA Profiles during Fetal Programming due to Maternal Overweight and Obesity. Gynecol. Obstet. Investig..

[128] Siddeek B., Mauduit C., Chehade H., Blin G., Liand M., Chindamo M., Benahmed M., Simeoni U. (2019). Long-term impact of maternal high-fat diet on offspring cardiac health: Role of micro-RNA biogenesis. Cell Death Discov..

[129] de Barros Sene L., Lamana G.L., Schwambach Vieira A., Scarano W.R., Gontijo J.A.R., Boer P.A. (2021). Gestational Low Protein Diet Modulation on miRNA Transcriptome and Its Target During Fetal and Breastfeeding Nephrogenesis. Front. Physiol..

[130] Sene Lde B., Mesquita F.F., de Moraes L.N., Santos D.C., Carvalho R., Gontijo J.A., Boer P.A. (2013). Involvement of renal corpuscle microRNA expression on epithelial-to-mesenchymal transition in maternal low protein diet in adult programmed rats. PLoS ONE.

[131] Sene L.B., Rizzi V.H.G., Gontijo J.A.R., Boer P.A. (2018). Gestational low-protein intake enhances whole-kidney miR-192 and miR-200 family expression and epithelial-to-mesenchymal transition in rat adult male offspring. J. Exp. Biol..

